# The Use of SenseWear Armband for Assessment of Daily Energy Expenditure and the Relation to Body Fat Distribution and Nutritional Intake in Lean Women with Polycystic Ovary Syndrome

**DOI:** 10.1155/2020/9191505

**Published:** 2020-05-06

**Authors:** Gulcan Arusoglu

**Affiliations:** Department of Nutrition and Dietetics, School of Health Sciences, Kirklareli University, Kirklareli, Turkey

## Abstract

**Objective:**

To evaluate nutritional intake, energy expenditure, and segmental body composition in lean women with polycystic ovary syndrome (PCOS) and compare them with age- and body mass index- (BMI-) matched control women.

**Methods:**

32 nonobese patients with PCOS and 31 age- and BMI-matched healthy women were included in the study. Energy expenditure and physical activity level were assessed by metabolic Holter equipment (SenseWear Armband, SWA) which was never previously used in lean PCOS population. Food intake is recorded with 24 hours of food record. Segmental body composition analysis was assessed by bioelectrical impedance analyses (BIA).

**Results:**

Mean BMI was 22.64 ± 3.64 and 21.55 ± 2.77 kg/m^2^ (*p*=0.185) in PCOS and control groups, respectively. Mean age was 22.03 ± 4.21 and 21.71 ± 2.67 year (*p*=0.720), respectively. No significant differences were found in total energy intake and percentage of carbohydrates, fats, and other micronutrients (*p* > 0.05). Energy percentage of proteins (%12.73 ± 1.98, *p*=0.008) was statistically lower in subjects versus the control group. The measurements of physical activity duration (PAD) (1.40 ± 0.87/2.18 ± 0.99 hours, *p*=0.002), active energy expenditure (372.35 ± 198.32/494.10 ± 186.50 kcal, *p*=0.018), and step counting (9370.03 ± 3587.49/11730.90 ± 3564.31 steps, *p*=0.013) measurement of the PCOS group were lower than the control group, respectively.

**Conclusions:**

New diagnosed women with PCOS had similar distribution and quantity of body fat parameters and nutritional status when compared to healthy women. Control subjects were found more active in energy expenditure.

## 1. Background

Polycystic ovary syndrome (PCOS) is the most common endocrine disorder of women of the reproductive age [1]. Different percentages were obtained in studies on the prevalence of polycystic ovary (PCO) with different definitions. According to the National Institute of Health (NIH), PCO is seen 4–8% worldwide. The incidence of the syndrome is higher according to the criteria of Rotterdam and the Association of Androgen Excess PCOS Society [2]. Because PCO is considered as a group of obese patients, these individuals are often neglected. Therefore, these patients should be carefully evaluated and treated. According to epidemiological data, there are those who are obese and nonobese in width: 20% in the Korean population and 27–50.5% in Europe, and 67% of PCOS in the US are obese. When it comes to different ethnic origins, the prevalence of women with PCOS who are in normal weight and underweight is reported to be 1.5–6.6%. As in our study, subjects with BMI below 25 kg/m^2^ are considered nonobese and BMI over 25 kg/m^2^ is considered obese [3]. However, although the majority of PCOS women is overweight or obese, many lean women with PCOS are also considered at high risk for metabolic disorders such as hyperinsulinemia and insulin resistance. Independent of BMI, this circumstance may lead to some abnormalities such as ovulatory dysfunction and polycystic ovaries and higher intra-abdominal fat [4].

Assessment of human body combination is an important factor for determining the dietary status of an individual and of a group in health and disease [5]. This is important for the evaluation and of the quantity and the distribution of fat mass [6]. Measuring body composition is more useful than measuring body weight gain or weight loss to determine actually whether muscle mass or body fat is increased or lost [6]. Measuring body composition distinguishes fat and fat-free mass which calculating BMI could not [7]. Fat mass differences changed with sex, ethnicity, and age which are influenced by genetic, nutritional status, diet, behaviour, and socioeconomic, environmental, and hormonal factors [6]. If compared with the control group, there is higher waist-to-hip ratio, greater intraperitoneal and visceral fat, and percentages of body fat in BMI-matched lean women with PCO [3]. Android fat pattern is the central in the cardiovascular risk management of metabolic syndrome. Android or central fat deposition is known to be more associated with cardiometabolic risk than gynoid or peripheral fat deposition which is more common in males than females [8]. This type of fat distribution is associated with hyperinsulinemia, impaired glucose tolerance, diabetes mellitus, and increased androgen production rates [9]. Gynoid adiposity is more common in females than men, but is less associated with cardiometabolic risk compared with android adiposity [10]. The android fat pattern has been described in obese and lean women with PCOS [9]. Together, there is conflicting data available about segmental body fat in women with PCOS [11, 12].

Studies do not investigate the diet constituents of especially lean PCOS patients. This study also aimed to compare nutritional intakes of lean women with PCOS to the healthy group. It is extremely important for women with PCOS who are lean to get a variety of macro- and micronutrients in their diet; therefore, they need to make sure that their diet contains plenty of vegetables and fruit [13]. The nutrient profile of individuals provides information on the relationship between nutritional intake, nutritional status, and health outcomes [14]. In addition, because lean patients with PCOS do not need to lose weight, they do not need to restrict their caloric intake [13]. Nutrient status in lean individuals with PCOS is also assessed for the first time in this study in our country. Lean women populations with PCOS are a unique group and have different phenotypic, metabolic, hematologic, and neurologic characteristics than obese participants with PCOS [13]. It is necessary to see the profile of Turkey. Some data show that women with PCOS have a nutritional target more prone to consume a carbohydrate-rich diet and a low-fiber diet than matched controls. This observation may be related to the default change of signals such as neuropeptide Y, ghrelin, leptin, and insulin which arise from the brain, intestine, pancreas, and adipose tissue and controls the nutritional behavior [15]. This study compared the nutritional intake of the two groups by using 24-h food record for 3 consecutive days (two weekdays and one weekend).

According to some authors, energy consumption performance could be naturally reduced in polycystic ovary syndrome [15]. Basal metabolic rate (BMR) and active energy expenditure (AEE) are components of total energy expenditure (TEE). AEE called as energy consumption in physical activity. The SenseWear Armband (SWA) is used as a practical tool recently for estimating total energy expenditure and active energy expenditure. Due to the experimental protocols or activities in each study, in the results of specific characteristics of each population and the monitor firmware or the specific characteristics of each population, there is a great heterogeneity for the validity and precision of the SWA in energy expenditure estimation [16]. According to Fruin and Rankin [17], this validated tool has many advantages in clinical use compared to traditional ways as it gives more comfort to patients, costs lower, and gives fast collection of data [15, 17]. SWA has not been used in lean PCOS patients so far.

To this purpose, the present study has investigated differences and the possible associations between nutritional intake in accumulation and distribution of body fat and energy expenditure with SWA equipment in lean women with PCOS and compared them with that of healthy group matched for age and BMI.

## 2. Materials and Methods

### 2.1. Subjects

32 subsequent subjects with polycystic ovary syndrome and 31 age- and BMI-matched healthy controls were collected for the study who attended the Endocrinology and Metabolism outpatient clinic of Hacettepe University between the year 2011 and 2013. All included women fulfilled the Rotterdam criteria [18].

Exclusion criteria were women with chronic diseases and presence of renal, type 2 diabetes, and cardiovascular or hepatic disease. Before enrollment to this study, none of the subjects ingested any medication for a duration of at least three months. Patients remained in their routine nutritional plan during the study.

### 2.2. Anthropometrics and Segmental Body Composition

Weight and height were measured with standard techniques. Women were weighed in underwear with a precision weighing of 0.1 kg, and height measurements were recorded with a precision measuring device up to 0.5 cm (Seca 703, Germany). BMI (weight (kilograms)/height (meters)^2^) was calculated. The BMI classification was made in accordance with the World Health Organization [19].

BMR (kcal), total fat mass (FM), total body water (TBW), fat-free mass (FFM), impedance (Ὡ), and segmental measures were predicted by using the BIA Segmental Body Analysis Monitor system (TANITA, BC-418 MA type). Units were calculated both in kilograms and percentages. BIA calculates and predicts the BMR. BIA is used as a method to conduct electricity between fat and water components, based on the conduction of electric current and differences in the body's ability. Before measurements, the subjects were instructed to avoid food intake for 4 h and strenuous exercise for 24 h. Measurements were made at room temperature, and the subjects were not allowed to wear metal objects. Dehydration and/or menstrual conditions were the exclusion criteria that changed the body fluid-electrolyte balance.

### 2.3. Energy and Nutrient Intake and Food Patterns

Questions were about general features, anthropometric measurements, and 3-day food consumption. All the questionnaires were conducted face-to-face by the researcher. Food intake record was assessed by a 24-h dietary record for 3 sequential days by using an Atlas of food photos showing portion sizes [20]. Dietary assessment was made at the beginning of the study. Dietary records for total micro- and macronutrient intake were analyzed with the software according to the standard food tables [21].

### 2.4. TEE

Parameters for total energy expenditure (TEE), physical activity duration (PAD), sleep duration, activity energy expenditure (AEE), average METs, and number of steps (NS) were determined for three days by SenseWear Armband (Body Media Inc., Pittsburg, PA, USA). Armband Metabolic Holter was used in order to determine the level of physical activity (PA) [22]. The subjects were given the SenseWear 6.1 brand Armband (SW-BodyMedia, Pittsburg, USA) for three sequential days on the left arm at each of the three phases in which food consumption was taken. Armband was not inserted during bath. Data are determined by the relevant software program, and the PA states of the individuals were determined. The temperature sensors on the armband detect the temperature changes in the body, the motion sensors detect the activity moment, and the stress sensors measure the skin stress. During the lifetime of the device, subjects did not participate in any PA other than mandatory daily activities. At the end of the measurement, average daily parameters were evaluated [23].

### 2.5. Statistical Analysis

NCSS (Number Cruncher Statistical System) 2007 (Kaysville, Utah, USA) was used for statistical analysis. Normal distribution of quantitative data and descriptive statistics was compared by Student's *t* test. Importance was assessed at *p* < 0.01 and *p* < 0.05.

## 3. Results

Age, height, weight, BMI, and BMR measures of the groups were insignificant (*p* > 0.05) (Table 1).

FM (%) measurements, TBW, and impedance measurements did not show statistically significant differences between the groups (*p* > 0.05). FM (kg) of the PCOS group was higher than the control group (*p*=0.050; *p* < 0.05). It was found that the FFM (kg) of the PCOS group was higher, but it was not statistically important (*p*=0.058; *p* > 0.05) (Table 2).

The right leg fat mass measurement of the PCOS group was higher when compared to the control group (*p* < 0.05). It was found that the PCOS group was also significantly higher in the right arm fat percentages (*p* < 0.05). Right arm fat mass measurement of the PCOS group was higher (*p* < 0.05) (Table 3).

Total energy expenditure, active energy expenditure, steps, sleep duration, average METs, inserting duration, and energy parameter measurements did not show statistically significant difference according to groups (*p* > 0.05). Physical activity duration measurement (*p* < 0.01), active energy expenditure measurement (*p* < 0.05), and step measurement of the PCOS group were significantly lower (*p* < 0.05) than the control (Table 4).

According to the groups, energy intake, water, protein, fat, carbohydrate, dietary fiber, and dietary cholesterol intake measurements did not show any important difference (*p* > 0.05). The percentage of protein consumed by the PCOS group was statistically lower than the compared group (*p*=0.008; *p* < 0.01) (Table 5).

Vitamin A, vitamin C, vitamin E, vitamin B_1_, vitamin B_2_, vitamin B_6,_ total folic acid, *β*-carotene, and Na, K, P, Ca, Mg, Fe, and zinc measurements did not show any statistically significance according to the groups (*p* > 0.05) (Table 6).

## 4. Discussion

PCOS is usually associated with overweight or obesity. This study evaluated nutritional intake, body composition, and energy expenditure of lean PCOS women and compared them with healthy controls. Nutritional status is assessed because the available literature does not provide the assessment of lean women with PCOS. Also, energy expenditure with SenseWear Armband is assessed for the first time in lean PCOS women in this country. SWA is a new and practical tool to measure energy expenditure [15].

Lean PCOS patients have limited data regarding segmental fat distribution in the literature [9]. It has been a reported in a study that women with PCOS who are lean had a higher amount of fat which was statistically important and a lower lean body mass than matched healthy controls [24]. Aydin et al. [11] and Good et al. [25] documented also that there were not any difference in body fat distribution and composition between the lean group with PCOS and healthy women [11, 25]. In this study, we did not find significant differences in distribution of fat between women with PCOS and controls. Right leg fat mass and right arm fat-free and fat mass showed significant higher measurements in PCOS than controls. More recent cross-sectional studies compared fat distribution by magnetic resonance imaging (MRI) between the lean group with PCOS and controls. The data have shown that lean patients with PCOS have less visceral fat when compared to control patients. Lean patients with PCOS had significantly lower subcutaneous adipose tissue development although height, weight, and BMI did not change significantly [13]. In particular, without change in weight, the body composition of women who exercise regularly may vary with increasing in lean body mass and low fat mass. Increased lean body mass increases resting energy consumption and can help to improve parameters such as hormonal and metabolic parameters in women with PCOS [26]. It has been observed that women with PCOs who are lean had lower caloric intake than healthy lean women, but found harder to maintain weight [13]. Our results indicate in this study that lean PCOs women had similar energy intake but significantly lower protein intake than controls (%12.73 ± 1.98; 14.39 ± 2.69, respectively). This result may mean that they ingest other macronutrients especially carbohydrates or fats higher than controls. Studies have shown there may not be an optimal diet or macronutrient composition for PCOS [4, 27]. It was revealed in a study by Nizareddin et al. (2018) that diet consumed by PCOS subjects was not a balanced one. Though intake of cereals, legumes, dairy products, meats, fats, and sugars were satisfactorily meeting the recommended dietary intake, unfortunately intake of roots, tubers, green leafy, and other vegetables and fruits were poorly met (Nizaruddin et al., 2018). On the contrary, it is reported in another study that there is no difference in macronutrient and energy intake between the two groups [28]. However, a study argued that the usual dietary macronutrient intake in women with PCOS did not show a difference in terms of the age-matched control group [15], and another study concluded that PCOS was affected by nutritional status [29].

In the present study, AEE measurement, PAD measurement, and step measurement of lean PCOS were lower than the control group. According to some research studies, there is no difference in energy or nutritional intake. Macro- or micronutrient intake or PA did not make a difference between the lean or overweight group with the PCOS or control [30]. Only one study which used SWA for the first time in PCOS population found resting metabolic rate measurements similar to the control group [15] which is similar to our data. In one of the PCOS studies, it was found that decreased BMR is shown, especially in women with insulin resistance. It is stated that these individuals should limit their energy intake and increase their energy expenditure by doing exercise to maintain their body weight [31]. Due to lack of available trials, it was not possible to compare energy consumption with SWA in lean patients.

## 5. Conclusions

PCOS in lean women is seen uniquely and have different phenotypic, metabolic, hematologic, and neurologic characteristics than obese participants with PCOS. In conclusion, nonobese women with PCOS had similar body composition compared to age- and BMI-matched healthy controls. Small sample size is the limitation of our study, and it cannot be extrapolated to all populations as it was conducted in a single research facility. As in any study that uses food record data, there is potential for inaccurate reporting. The results in this study emphasizes no differences between dietary intake; however, protein consumption was significantly lower in PCOS patients [32].

## Figures and Tables

**Table 1:** Evaluation of some anthropometric measurements of PCOS and the control group.

Variables	Groups	Mean ± SD	Min-max	Median	^a^ *p*
Age (year)	PCOS	22.03 ± 4.21	17–34	21	**0.720**
	Control	21.71 ± 2.67	18–33	21	

Height (cm)	PCOS	162.70 ± 7.10	149–183	164	**0.415**
	Control	161.42 ± 5.13	154–174	161	

Weight (kg)	PCOS	60.10 ± 10.43	42.5–90.7	59.9	**0.069**
	Control	56.01 ± 6.68	42.7–72.1	55.2	

BMI (kg/m^2^)	PCOS	22.64 ± 3.64	16.6–29.5	21.2	**0.185**
	Control	21.55 ± 2.77	17–27.4	21	

BMR (kcal)	PCOS	1345.85 ± 110.68	1112–1558	1325	**0.103**
	Control	1304.81 ± 85.03	1096–1508	1292	

^a^Student's *t*-test.

**Table 2:** Total body composition analyses.

Variables	Groups	Mean ± SD	Min-max	Median	^a^ *p*
FM (%)	PCOS	27.23 ± 6.9	15.7–40.1	27.4	0.089
	Control	24.53 ± 5.51	14.5–37	24.2	

FM (kg)	PCOS	16.85 ± 6.76	7.3–32	15.9	**0.050^∗^**
	Control	13.99 ± 4.69	7.1–24.7	13	

FFM (kg)	PCOS	42.95 ± 3.66	34.6–49	42.1	0.058
	Control	40.85 ± 4.85	18.8–48	41.3	

TBW (kg)	PCOS	40.68 ± 53.62	25.3–339	30.8	0.300
	Control	30.61 ± 2.14	24.9–35.7	30.5	

Impedance (Ω)	PCOS	705.63 ± 76.27	550–855	682.5	0.427
	Control	720.81 ± 74.26	578–929	709	

^a^Student's *t-*test, ^*∗*^*p* < 0.05, FM : fat mass, FFM : fat-free mass, and TBW : total body water.

**Table 3:** Segmental body composition measurements.

Measurements	Groups	Mean ± SD	Min-max	Median	^a^ *p*
RL_FM (%)	PCOS	32.01 ± 5.25	23.4–40.2	32.9	0.070
	Control	29.88 ± 3.76	22.6–37.7	29.6	
RL_FM (kg)	PCOS	3.59 ± 1.05	2.1–5.7	3.5	**0.035^∗^**
	Control	3.12 ± 0.65	1.9–4.4	3	
RL_FFM	PCOS	7.43 ± 0.66	6–8.8	7.35	0.206
	Control	7.23 ± 0.52	5.9–8.5	7.1	
LL_FM (%)	PCOS	31.75 ± 5.53	22.5–40	32.1	0.150
	Control	30.04 ± 3.56	23–36.9	29.4	
LL_FM (kg)	PCOS	3.5 ± 1.06	2–5.5	3.4	0.231
	Control	3.19 ± 0.97	1.9–7.1	2.9	
LL_FFM (kg)	PCOS	7.3 ± 0.66	5.9–8.8	7.2	0.080
	Control	7.04 ± 0.48	5.8–8.3	6.9	
RA_FAT (%)	PCOS	28.44 ± 7.86	13.7–44.9	26.9	0.035^∗^
	Control	24.02 ± 8.47	0.9–37.5	24.3	
RA_FM (kg)	PCOS	0.85 ± 0.38	0.30–1.80	0.75	0.249
	Control	0.74 ± 0.32	0.30–1.90	0.80	
RA_FFM (kg)	PCOS	2.01 ± 0.22	1.5–2.5	2	0.040^∗^
	Control	1.91 ± 0.15	1.5–2.2	1.9	
LA_FAT (%)	PCOS	29.37 ± 7.71	14.8–43.7	28.65	0.070
	Control	25.81 ± 7.59	0.9–38.6	25.8	
LA_FM (kg)	PCOS	0.89 ± 0.41	0.3–1.9	0.75	0.212
	Control	0.77 ± 0.34	0.3–1.9	0.7	
LA_FFM (kg)	PCOS	2.01 ± 0.26	1.4–2.5	2	0.070
	Control	1.91 ± 0.18	1.4–2.3	1.9	
TRUNK_FAT (%)	PCOS	23.42 ± 8.37	9.2–40	22.75	0.126
	Control	20.31 ± 7.5	7.2–42.1	19.1	
TRUNK_FM (kg)	PCOS	7.9 ± 4.02	2–17.4	6.6	0.080
	Control	6.35 ± 2.8	1.9–13.5	5.5	
TRUNK_FFM (kg)	PCOS	24.16 ± 2.04	19.8–28.4	23.7	0.075
	Control	23.31 ± 1.68	18.5–26.7	23.1	

^a^Student's *t-*test, ^*∗*^*p* < 0.05, RL : right leg, LL : left leg, RA : right arm, and LA : left arm.

**Table 4:** Armband metabolic Holter equipment.

Variable	Groups	Mean ± SD	Min-max	Median	^a^ *p*
TEE (kcal)	PCOS	2128.84 ± 289.91	1698–2880	2100	0.957
	Control	2124.66 ± 314.66	831–2502	2158	
PAD (hour)	PCOS	1.40 ± 0.87	0.16–3.33	1.23	**0.002^∗∗^**
	Control	2.18 ± 0.99	0.12–4.24	2.08	
AEE (kcal)	PCOS	372.35 ± 198.33	70–783	342	**0.018^∗^**
	Control	494.1 ± 186.5	48–892	507	
Steps	PCOS	9370.03 ± 3587.49	4004–19550	8583	**0.013^∗^**
	Control	11730.9 ± 3564.31	3200–19550	12191	
Sleep duration (hour)	PCOS	5.67 ± 1.23	2.43–7.58	5.52	0.185
	Control	6.09 ± 1.19	2.43–8.35	6.26	
Average METs	PCOS	1.49 ± 0.17	1.2–1.9	1.50	0.115
	Control	1.78 ± 1.01	1.2–7	1.60	
On-body duration (h)	PCOS	22.55 ± 2.04	15.27–24	23.45	0.581
	Control	22.21 ± 2.71	15.29–24	23.39	

^a^Student's *t-*test, ^*∗∗*^*p* < 0.01, ^*∗*^*p* < 0.05, TEE : total energy expenditure, PAD : physical activity duration, AEE : average energy expenditure, and METs : metabolic equivalents.

**Table 5:** Daily energy intake and macronutrient components of PCOS and control.

Macronutrients	Groups	Mean ± SD	Min-max	Median	^a^ *p*
Energy intake (kcal)	PCOS	1907.78 ± 559.71	1176–3431	1788.21	**0.484**
	Control	1814.25 ± 476.36	993–2845	1751.45	
Water (g)	PCOS	1582.82 ± 606.21	426.3–3159.82	1525.38	**0.946**
	Control	1595.13 ± 785.33	436.61–3345.1	1290.49	
Total protein (g)	PCOS	58.45 ± 15.38	32.36–101.52	57.04	**0.311**
	Control	62.29 ± 13.96	30.09–86.57	63.21	
Total protein (%)	PCOS	12.73 ± 1.98	9–16	12.5	**0.008^∗^**
	Control	14.39 ± 2.69	10–19	14	
Total fat (g)	PCOS	83.51 ± 34.74	34.22–205.62	72.84	**0.421**
	Control	77.28 ± 24.58	43.8–137.55	73.43	
Total fat (%)	PCOS	38.1 ± 6.2	26–53	37	**0.906**
	Control	37.9 ± 6.68	27–50	37	
Total CHO (g)	PCOS	225.43 ± 61.07	147.76–389.99	205.64	**0.454**
	Control	213.19 ± 65.74	90.55–346.6	195.95	
Total CHO (%)	PCOS	49 ± 6.26	35–59	49	**0.449**
	Control	47.77 ± 6.3	35–59	46	
Dietary fiber (g)	PCOS	20.55 ± 7.21	11.56–38.02	18.45	**0.498**
	Control	21.76 ± 6.67	8.28–34.48	20.41	
PUFA (g)	PCOS	21.58 ± 12.65	5.44–61.27	18.49	**0.605**
	Control	20.07 ± 9.83	3.29–47.93	19.83	
Dietary cholesterol (mg)	PCOS	212.97 ± 81.26	46.54–411.6	212.32	**0.624**
	Control	225.07 ± 108.35	98.67–606	207.82	

^a^Student's *t*-test, ^*∗∗*^*p* < 0.01.

**Table 6:** Daily micronutrient composition of PCOS and control.

Micronutrients	Groups	Mean ± SD	Min-max	Median	^a^ *p*
Vit A (mg)	PCOS	1231.37 ± 1314.54	343.02–7292.16	783.11	**0.766**
	Control	1153.14 ± 613.51	524.97–2638.29	933.59	
*β*-Carotene (mg)	PCOS	3.09 ± 2.76	0.45–15.13	2.44	**0.858**
	Control	3.21 ± 2.51	0.63–11.07	2.49	
Vit E eq (mg)	PCOS	21.93 ± 11.08	6.9–55.19	20.15	**0.355**
	Control	19.5 ± 9.23	5.24–51.86	19.9	
Vit B_1_ (mg)	PCOS	0.82 ± 0.31	0.48–2.11	0.74	**0.771**
	Control	0.85 ± 0.24	0.37–1.3	0.82	
Vit B_2_ (mg)	PCOS	1.1 ± 0.25	0.67–1.59	1.07	**0.068**
	Control	1.25 ± 0.36	0.62–2.07	1.14	
Vit B_6_ (mg)	PCOS	1.29 ± 0.34	0.81–2.1	1.26	**0.126**
	Control	1.55 ± 0.85	0.81–5.6	1.35	
Total FA (mg)	PCOS	280.41 ± 82.82	175.26–561.27	275.87	**0.489**
	Control	295.95 ± 90.99	180.31–471.01	279.53	
Vit C (mg)	PCOS	99.09 ± 41.29	37.47–229.34	85.95	**0.114**
	Control	117.3 ± 47.19	13.25–217.79	117.9	
Na (mg)	PCOS	1598.15 ± 627.72	564.71–2584.83	1456.11	**0.332**
	Control	1782.13 ± 823.86	791.41–4653.74	1742.96	
K (mg)	PCOS	2215.3 ± 560.02	1251.17–3348.04	2092.56	**0.079**
	Control	2485.33 ± 617.79	1524.26–3730.24	2357.49	
Ca (mg)	PCOS	599.83 ± 187.51	281.67–996.61	565.92	**0.176**
	Control	671.23 ± 217.76	351.75–1131.76	629.73	
Mg (mg)	PCOS	254.51 ± 92.81	150.51–526.8	232.48	**0.793**
	Control	260.42 ± 82.1	138.81–493.72	245.42	
P (mg)	PCOS	956.58 ± 267.85	481.5–1689.12	940.92	**0.246**
	Control	1036.57 ± 264.9	438.32–1626.59	1025.1	
Fe (mg)	PCOS	10.42 ± 3.07	6.21–18.42	9.58	**0.210**
	Control	11.49 ± 3.51	5.12–19.38	11.16	
Zinc (mg)	PCOS	8.91 ± 2.67	5.25–15.75	8.59	**0.452**
	Control	9.41 ± 2.51	3.87–14.72	9.19	

^a^Student's *t*-test.

## Data Availability

The data used to support the findings of this study are included within the article.
